# Unraveling the Compositional and Molecular Features Involved in Lysozyme-Benzothiazole Derivative Interactions

**DOI:** 10.3390/molecules26195855

**Published:** 2021-09-27

**Authors:** Ramón Rial, Michael González-Durruthy, Manuel Somoza, Zhen Liu, Juan M. Ruso

**Affiliations:** 1 Soft Matter and Molecular Biophysics Group, Department of Applied Physics, University of Santiago de Compostela, 15782 Santiago de Compostela, Spain; ramon.rial@usc.es (R.R.); michael.durruthy@usc.es (M.G.-D.); manuel.somoza@usc.es (M.S.); 2 Department of Chemistry and Biochemistry, LAQV@REQUIMTE, Faculty of Sciences, University of Porto, 4169-007 Porto, Portugal; 3 Department of Physics and Engineering, Frostburg State University, Frostburg, MD 21532, USA; zliu@frostburg.edu

**Keywords:** lysozyme, BTS, molecular docking, protein interactions, ITC

## Abstract

In this work we present a computational analysis together with experimental studies, focusing on the interaction between a benzothiazole (BTS) and lysozyme. Results obtained from isothermal titration calorimetry, UV-vis, and fluorescence were contrasted and complemented with molecular docking and machine learning techniques. The free energy values obtained both experimentally and theoretically showed excellent similarity. Calorimetry, UV-vis, and 3D/2D-lig-plot analysis revealed that the most relevant interactions between BTS and lysozyme are based on a predominance of aromatic, hydrophobic Van der Waals interactions, mainly aromatic edge-to-face (T-shaped) π-π stacking interactions between the benzene ring belonging to the 2-(methylthio)-benzothiazole moiety of BTS and the aromatic amino acid residue TRP108 of the lysozyme receptor. Next, conventional hydrogen bonding interactions contribute to the stability of the BTS-lysozyme coupling complex. In addition, mechanistic approaches performed using elastic network models revealed that the BTS ligand theoretically induces propagation of allosteric signals, suggesting non-physiological conformational flexing in large blocks of lysozyme affecting α-helices. Likewise, the BTS ligand interacts directly with allosteric residues, inducing perturbations in the conformational dynamics expressed as a moderate conformational softening in the α-helices H1, H2, and their corresponding β-loop in the lysozyme receptor, in contrast to the unbound state of lysozyme.

## 1. Introduction

Proteins are the base of cellular processes because they play an essential role in the transportation and deposition of endogenous and exogenous substances, such as hormones, fatty acids, and medicinal drugs. Consequently, understanding their structure, functions and interactions are major tasks [1,2,3,4,5].

Lysozyme, a very popular protein that has become a model system in protein chemistry, enzymology, crystallography, and molecular biology, was discovered by Alexander Fleming in 1921, by observing how a nasal mucus drop of a cold he had accidentally fell onto a sample with bacteria. That action caused the lysis of bacteria and led him to discover a “remarkable bacteriolytic element”, which he later called lysozyme [6]. Lysozyme is present in various biological fluids and tissues, including saliva, tears, skin, liver blood, and lymphatic tissues of humans and other animals [7]. It has been widely used in the pharmaceutical and food fields because of its functions, such as anti-inflammatory, anti-viral, immune modulator, anti-histaminic, and anti-tumor activities [8,9]. Moreover, lysozyme is a model system in protein chemistry, enzymology, crystallography, and molecular biology. In functional fields, there are many aspects concerning the biological role of lysozyme. As said before, lysozyme is a protein which takes part in the first barrier of defense. Researches have shown that this protein is involved in the innate immune system and can protect cells from death by lysing the cytoderm of bacteria [10,11].

Structurally, lysozyme is a tiny, monomeric, globular enzyme with low molecular weight (14.4 kDa). It has common elements which appear in other proteins, such as alpha helices and beta sheets. In addition, it is formed by 129 tactic amino acid residues containing six tryptophan (trp), three tyrosine (tyr), and four disulfide bridges. In terms of properties, lysozyme has high stability and structure. This makes lysozyme an excellent macromolecule for studying the influence of several interactions with other elements on its activity [12].

Furthermore, its high-resolution crystal structure presents two dominant fluorophores close to the substrate-binding site. They are Trp62 and Trp108 and are important in the union function or stabilizing the structure, either by substrate or by inhibitor. Therefore, using these tryptophan residues, lysozyme gives the possibility of correlating its dynamics with enzymatic activity by means of fluorescence analyses. This technique could provide relevant information on the interaction between lysozyme and a ligand, and the change around the union area that a ligand could produce [13].

Another relevant function of lysozyme is its ability to carry drugs. The effectiveness of drugs, most of their cellular signaling, and cellular metabolic pathways depend on their union. Due to this, there has been a large amount of research studies to figure out the union interactions between protein and drug [14]. Therefore, the studies focused on obtaining information on how protein-drug interactions work are vital due to their enormous potential application. Curiously, the interactions between proteins and several small molecules, such as metal ions, non-metal ions, and pharmaceutical elements have been widely studied [15,16,17], but the interaction in which lysozyme has the protein role are less abundant. The development of new anti-tumor drugs is a constant in the health sciences; the number of them is growing exponentially. Several studies have shown that the benzothiazole nucleus possesses potent anticancer activity against human cancer. In fact, molecules with the benzothiazole core have been shown to have the ability to kill cells in a tumor-specific manner. Benzothiazole derivatives have been reported to possess potent anti-cancer properties due to their structural similarity to natural purines, as they can readily interact with biomolecules in living systems [18]. From a theoretical point of view, the structure of BTS is very useful for investigating the nature of the interactions of aromatic amino acid residues with this molecule. This is because the orientation presents great similarities with those found in complexes formed between aromatic molecules such as benzene, where the stabilization of this type of system has been rationalized based on models involving quadrupolar interactions [19]. Moreover, 3-(2-Benzothiazolylthio)-propanesulfonic acid (BTS) has been reported as a powerful and selective inhibitor of triosephosphate isomerase from Trypanosoma cruzi, the parasite that causes the Chagas disease [20]. In a previous work, we analyzed the interactions between BTS and lysozyme by differential scanning calorimetry and circular dichroism measurements [21]. Based on the data obtained, we conclude that in the binding process electrostatic interactions are present, and high concentrations of BTS (>0.25 mM) are necessary to denature the protein. Whether any other interactions are present, or possible conformational changes are occurring at low concentrations of BTS remains unresolved.

Despite the many studies carried out to date, the unknowns about this type of system remain open. However, recently experimental techniques have been reinforced by the progress that computational tools have undergone, thus allowing the contrast of results obtained and offer new interpretations [22,23,24]. The most recent of these techniques have been a set of algorithms known as Machine Learning, which are allowing considerable savings in computational time. Based on the above, the objective of this work is to offer a new vision of the interaction process between lysozyme and BTS from an experimental and computational point of view that allows us to understand this important process in a clear and precise way.

## 2. Results and Discussion

### 2.1. Experimental Procedures

From an experimental perspective, the interaction of the ligand and the macromolecule can be characterized by means of the isothermal titration calorimetry (ITC) technique. Small molecules are bound to macromolecules by four binding modes: Van der Waals, hydrogen bonds, electrostatic, and hydrophobic forces [25]. According to enthalpy (ΔH°) and entropy (ΔS°) changes, the interaction type can be determined. For ΔH° and ΔS° > 0, the main interaction mode is led by hydrophobic forces; for ΔH° and ΔS° < 0, the main responsible forces are Van der Waals interactions and hydrogen bonds, and for ΔH° < 0 and ΔS° > 0, electrostatic forces [26].

**Figure 1:**
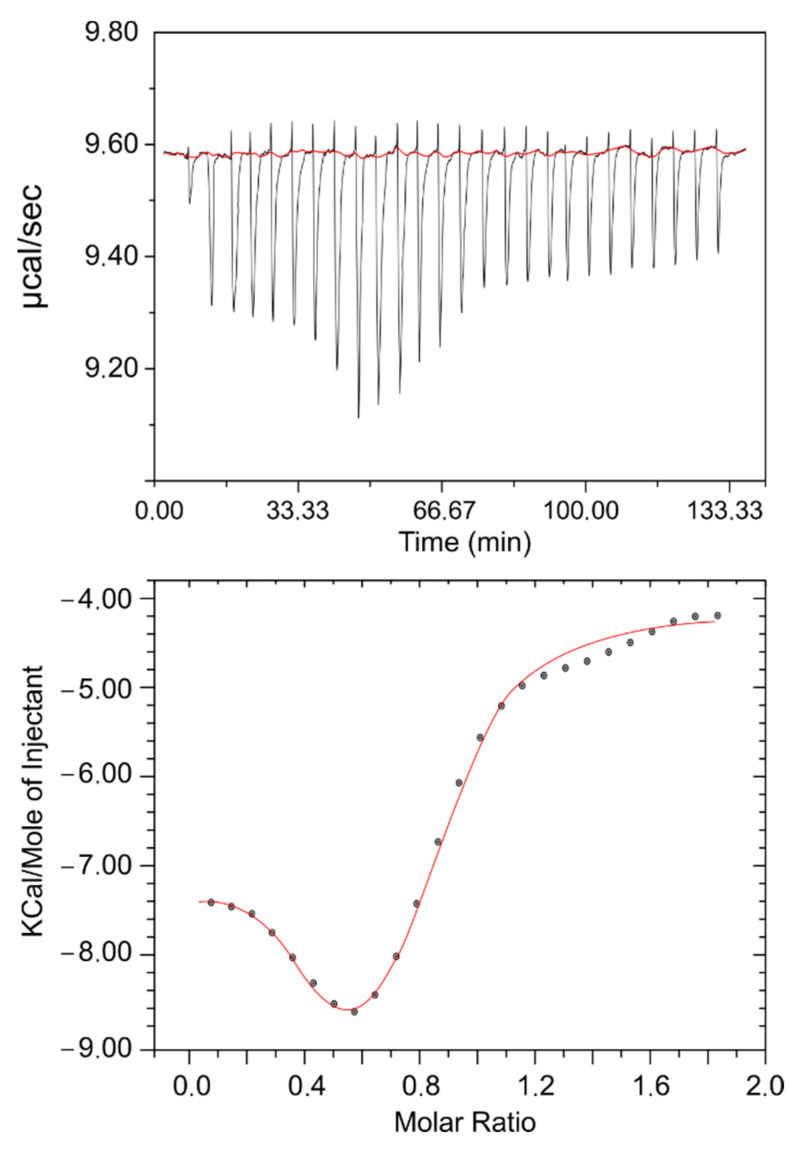
Heat of interaction. ITC results for the titration of the BTS drug (0.5 mM) into the lysozyme solution (0.02 mM) at 298.15 K. Each dot represents an injection. The heat of the first injection was neglected to get a more accurate fitting.

Typically, in this type of test, a protein is inserted in the calorimetric cell and a ligand is injected through a syringe needle at the desired rate. In the present case, the initial concentrations used were 0.02 mM for the protein and 0.5 mM for the drug, assuring a final ratio high enough to reach saturation. Figure 1 shows the results relating the amount of heat released per injection and the molar ratio (drug/protein). At first glance, a few simple deductions can initially be made: an interaction exists; it is exothermic and gradually decreases as the drug/protein ratio increases; and saturation is found to be reached at molar ratios higher than one. With the designed experimental configuration, equilibrium is roughly reached after 100 min. For further analysis, the thermodynamic parameters were calculated using the Van ‘t Hoff equation:

ln K = −ΔH°/RT + ΔS°/R(1)

where R is the molar gas constant, T is the experimental temperature, and K is the association constant.

Data were fitted to a single-site binding model. The binding stoichiometry (n), binding constant (K), enthalpy change (ΔH°), and entropy change (ΔS°) were obtained from the results of the fitting and the values obtained were as follows: n = 0.95 ± 0.14; K = (3.82 ± 0.79) × 10^5^ M^−1^; ΔH° = −113.8 ± 24.1 kJ mol^−1^; and ΔS° = −0.29 ± 0.07 kJ mol^−1^ K^−1^. The stoichiometry reveals that only one type of binding takes place, while the binding constant denotes that the BTS is a rather moderate binder. For its part, negative values of entropy and enthalpy changes indicate that the hydrogen bonding and Van der Waals forces play a major role in the interaction between the drug and the biomacromolecule, suggesting a loss of conformational freedom related to the formation of the complex [27].

When BTS binds to some of the proteins under study, it acquires an induced circular dichroism (CD) spectrum through chiral perturbation to its structure or electron rearrangements. The intensity of the CD spectrum is determined by the strength of the interactions and both the geometry of the BTS molecule and the residue in the protein backbone. Thus, the CD can be used to probe the binding of BTS molecules to the proteins under study, providing information about the secondary structure of the protein. Table 1 shows the changes in the α-helices and β-sheets content of lysozyme at different BTS concentrations [21]. In general, the presence of BTS does not produce major changes in the protein structure and it can be seen that the β-sheets content is always greater than the α-helices content. α-helices are more sensitive to the presence of BTS, decreasing their content by 37% as the concentration of the drug increases. On the other hand, β-sheets only decrease their content by 17%. These results show that at low concentrations, BTS does not induce important changes in the structure of lysozyme that could alter its physiological behavior.

**Figure 2:**
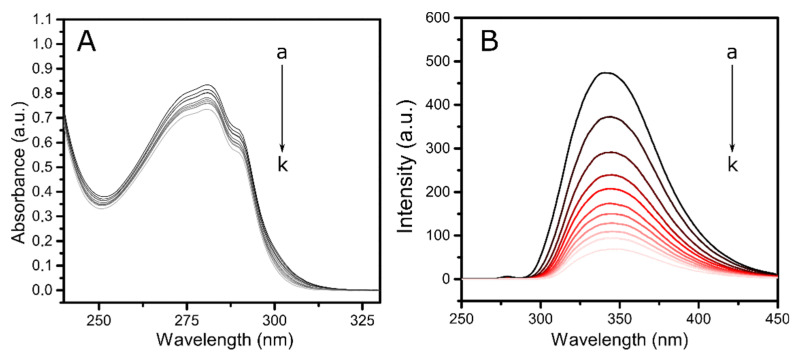
(**A**) UV absorption spectra of lysozyme in the absence and presence of BTS: C_lysozyme_ = 0.02 mM; C_BTS_ (×10^−3^) (a–k) = (0, 1, 2, 3, 4, 5, 6, 7, 8, 9, 10) mM. (**B**) Fluorescence emission spectra of lysozyme in the absence and presence of different concentrations of BTS at λ_ex_ = 280 nm: T = 298 K; C_lysozyme_ = 0.02 mM; C_BTS_ (×10^−2^) (a–k) = (0, 2, 4, 6, 8, 10, 12, 14, 16, 18, 20) mM.

UV-visible absorption spectroscopy is a very useful tool for assessing conformational alterations in protein-ligand systems. Analyzing the spectral curves under different conditions, the formation of a complex between the biomacromolecule and the drug can be decoded, as well as give insight into the consequential structural changes induced. In the case of lysozyme, the main aromatic amino acid residues that are responsible for the absorption peak at 280 nm are tyrosine, phenylalanine, and tryptophan, with tryptophan being the major contributor in the spectra [14]. Figure 2A shows the spectral curves of pure lysozyme and different systems of protein-ligand with increasing concentrations of BTS. In this case, the peak at 280 nm slightly decreases with the addition of BTS with no obvious drug shift, suggesting an increase in the hydrophobicity of the microenvironment around the aromatic amino acids of the biomacromolecule [28].

To obtain further information about the drug-protein interactions, fluorescence measurements were conducted. From previous studies [29,30] it is known that lysozyme presents a moderately rigid structure, which includes α-helices, β-sheets, and turns and loops. It is composed of 129 amino acid residues, including six Trp, four disulfide bridges, and three Tyr residues. The fluorophores with a major contribution are Trp-62 and Trp-108, both of them situated at the substrate-binding sites [31]. Usually, protein fluorescence is excited at wavelengths close to 280 nm or higher. The absorption at this maximum is caused by both Trp and Tyr, while at 300 nm or longer, the protein adsorption is essentially due to Trp. The reduction in the quantum yield of fluorescence from a fluorophore is known as quenching. It can be caused by a series of molecular interactions with a quencher molecule, such as energy transfer, excited-state reactions, ground-state complex formation, molecular rearrangements, and collisional quenching processes [32]. From the analysis of the spectra, some valuable information on the binding of proteins to simple molecules or even to small particles can be obtained [33]. In this work, the binding mechanism, constant, binding site, and intermolecular distance were obtained by analyzing the intrinsic fluorescence of pure lysozyme with the addition of different concentrations of the BTS drug. Preliminary experiments were performed to remove any possible imprecision provoked by the existence of inner filter effects. This phenomenon appears when the ligand causes absorption during excitation and emission radiation processes [28]. In this study, the inner filter effect was corrected following the procedure explained in the Materials and Methods section. Figure 2B pictures the emission spectra of lysozyme in the presence of increasing concentrations of BTS. As it can be observed, when excited with a wavelength of 280 nm, the protein alone presents a great emission band at 340 nm which is progressively reduced with no shift as the drug is added. This fact signifies the beginning of saturation of the lysozyme binding sites [30] and, at the same time, indicates that the microenvironment of tryptophan is relatively less hydrophobic upon binding with the drug.

For studying the fluorescence quenching, data is typically analyzed through the well-known Stern–Volmer equation which can classify the type of fluorescence quenching process as static or dynamic:

F_0_/F = 1 + k_q_τ_0_[Q] = 1 + K_sv_[Q] (2)

where F_0_ and F are the steady-state fluorescence intensities before and after the addition of the quencher, K_sv_ is the Stern–Volmer quenching constant, [Q] corresponds to the concentration of the quencher (in this case, BTS), k_q_ is the quenching rate constant of the protein, and τ_0_ is the average lifetime of the biomolecule, taken as 1.8 × 10^−9^ s for lyz [34].

**Figure 3:**
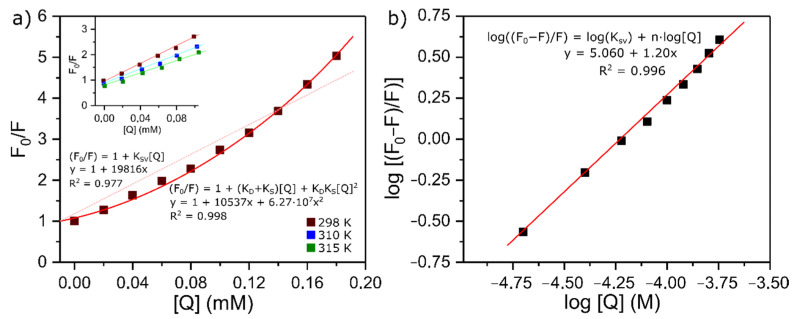
(**a**) Stern–Volmer plots for the quenching of lysozyme by BTS. Insert: Quenching at low concentrations and different temperatures (298, 310, 315 K); C_lysozyme_ = 0.02 mM. (**b**) Plots of log[(F_0_ − F)/F] vs. log [Q]. Numerical results of the fittings are presented in the graphs.

A Stern–Volmer plot of F_0_/F against [Q] is linear for purely dynamic (K = 0) or purely static (k = 0) quenching, but curved upwards for combined quenching (eqn (3)). On this basis, a curvature of a Stern–Volmer plot is often taken to indicate combined quenching. The plot obtained for this case is shown in Figure 3a. It deviates significantly from linearity, which indicates that there is a coexistence of interactions occurring between lysozyme and BTS when the protein is in its ground electronic state and in its first excited single state: static and dynamic quenching, respectively [34]. Taking into consideration that the intensity ratio F_0_/F is given by the product of static and dynamic quenching, Equation (2) would be more accurate as follows:

F_0_/F = (1 + K_S_[Q])(1 + K_D_[Q]) = 1 + (K_D_ + K_S_)[Q] + (K_D_K_S_)[Q]^2^ (3)

where K_S_ and K_D_ represent the dynamic and static quenching constants. As it can be seen in Figure 3a, at high concentrations of the quencher, the Stern–Volmer plot follows a continuous ascending curve. In this case, a quadratic least square fit was found to be adequate, with a correlation constant very close to 1 (R^2^ = 0.998). However, in the insert are the results for low concentrations of the drug, ranging from 2.0 × 10^−5^ to 1.0 × 10^−4^ L mol^−1^, and it can be observed that they present a good linear relationship (R^2^ ≥ 0.98). The K_SV_ obtained from the slope at a low concentration and 288 K was (1.66 ± 0.01) × 10^−4^ L mol^−1^ and it descends with an increase in temperature. Therefore, it can be inferred that the interaction between lysozyme and BTS is a static quenching process resulting from the formation of a lysozyme-BTS complex.

This assumption can be further confirmed from the calculated value of k_q_. In the present case, this constant has a value of 9.22 × 10^12^ L M^−1^ s^−1^ and is much greater than the value of the highest scatter collision quenching constant (2.0 × 10^10^ M^−1^ s^−1^) for different ligands with proteins [35]. Gathering this information, it is safe to assume that, at low concentrations, the probable quenching mechanism involves a complex formation, but the upwards curvature shown in Figure 3a suggests that at higher concentrations the quenching process is a result from a combined one.

Apart from the mechanisms involved, fluorescence measurements are also useful to get quantitative information about the binding. It has been previously demonstrated that if there are independent and similar binding sites in the biomolecule, both the number of binding sites, n, and the binding constant, K_A_, can be determined using the following equation [36]:

log [(F_0_ − F)/F] = log K_A_ + n · log[Q] (4)

The number of binding sites per molecule of lysozyme and the binding constant can be determined by the slope and the intercept of the double logarithm regression curves (Figure 3b). Linear fitting of experimental data gives values of (1.20 ± 0.14) and (1.15 ± 0.06) × 10^5^ M^−1^ for n and K_A_, respectively. Such results indicate that there is only one independent class of binding sites between the protein and the ligand, and the binder has a reasonable affinity for the protein, reinforcing the conclusions previously drawn from the isothermal titration calorimetry assays.

A more complete description of the interaction can be obtained by the analysis of the energy transfer, which can be used to find the distance between the tryptophan residues in the protein. According to the well-known Förster’s nonradioactive energy transfer theory (FRET), the energy transfer takes place when the fluorescence emission spectrum of the donor and UV-vis absorbance spectrum of the acceptor overlap considerably and the distance between donor and receptor is less than 8 nm [37,38]. In the present case, the donor is lysozyme and acceptor is BTS, and there is a significant overlap between emission and absorption spectra. Based on Förster’s theory, the efficiency of energy transfer (E) can be related to the distance between the donor and acceptor (r) as follows:

(5) $$E=1-(F/F_{0})=R_{0}^{6}/(R_{0}^{6}+r^{6})$$

where F and F_0_ are the fluorescence intensities of the protein in the presence and absence of BTS, respectively, and R_0_ is the critical distance when the energy transfer efficiency is 50%. This value can be calculated as follows:

(6) $$R_{0}^{6}=8.79\times 10^{-25}K^{2}n^{-4}\Phi J$$

where K^2^ is the spatial orientation factor of the dipole, n is the refractive index of the medium, Φ is the fluorescence quantum yield of the donor, and J is the effect of the overlap between the emission spectrum of lysozyme and absorption spectrum of BTS. The dipole orientation factor is the most imprecise value when calculating R_0_. Its value can theoretically be within the range of 0 to 4, but assuming that both the protein and the ligand are tumbling rapidly and free to take any orientation, then K^2^ equals 2/3. J, for its part, can be obtained by the equation:

J = (∑ F(λ) ε(λ) λ^4^ Δλ)/(∑F(λ) Δλ) (7)

where F(λ) is the fluorescence intensity of lysozyme at wavelength λ and ε(λ) is the molar absorption coefficient of BTS at wavelength λ. Solving the respective equations with the collected experimental data, the values of E, J, and R_0_ can be calculated, and consequentially, the distance between the donor and acceptor (r), can be determined as well.

**Figure 4:**
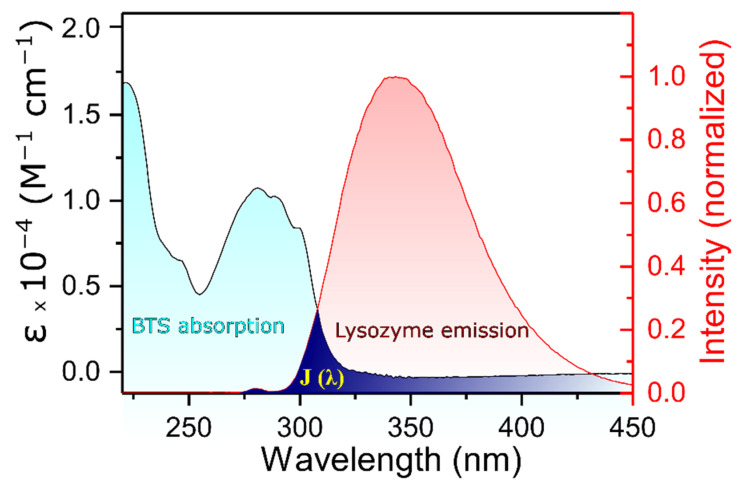
The overlap (dark blue) of the fluorescence emission spectrum of lysozyme (red) and the absorption spectrum of BSA (light blue) (*T* = 298 K); C_lysozyme_ = C_BTS_ = 0.02 mM.

Figure 4 shows the spectral overlap J when the molar ratio of the acceptor and donor is 1:1 and at 298 K. The value obtained for the integral was calculated to be equal to 1.26 ×10^13^ M^−1^ cm^−1^ nm^4^. Taking into consideration that the corresponding values of the parameters used were K^2^ = 2/3, Φ = 0.15, and n = 1.36 [39], R_0_ and r can be obtained by combining Equations (5) and (6). In this case, E = 0.21, R_0_ = 1.78 nm, and r = 2.22 nm, which means that the distance between the donor and acceptor (r) is lower than the maximal academic critical distance for R_0_ (5–10 nm) and is within the range of 2–8 nm. Furthermore, 0.5 R_0_ < r < 1.5 R_0_, clearly suggesting that the energy transfer from lysozyme to the drug occurs with a high probability, again reinforcing the idea that the quenching mechanism involved corresponds to a static one [30].

### 2.2. Computational Procedures

**Figure 5:**
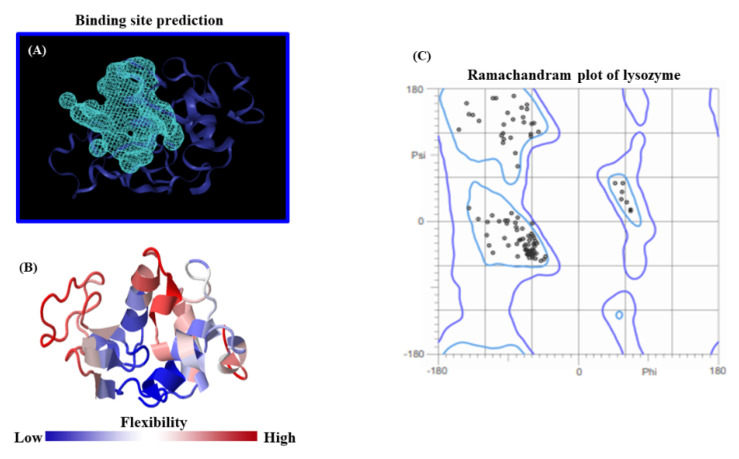
(**A**) Top view of the *fpocket* prediction for the best-ranked lysozyme binding site crystallographically validated by Deepsite software considering its position in the lysozyme. Herein, the obtained maximum cavity volume was equal to 431.26 Å^3^ (i.e., represented here by the light blue mesh-volumetric Van der Waal map) which involves the binding residues interacting with the BTS-ligand. At the bottom, (**B**) represents the lysozyme flexibility profile as a 3D-colored structure, according to the size of fluctuations driven by the whole structure from regions with low-flexibility (blue) to high-flexibility (red). In the right, (**C**) corresponds to the obtained Ramachandran plot for all the possible combinations of Psi vs. Phi dihedral torsion angles of the modeled lysozyme protein. Here, the area inside the Ramachandran contour line (purple color) shows the spatial distribution of conformationally favored residues (black dots), while the outlier or conformationally unfavored non-binding residues are fully absent for this instance.

One of the most critical steps to ensuring the quality of modeling results is the accurate prediction of feasible lysozyme binding-sites, coupled with an appropriate crystallographic structural validation of this receptor, and its flexibility properties. Herein, the search space was set up for the lysozyme receptor by using the ezPocket tool with the Deepsite validation procedure [40,41,42], as a cubic grid box with a size of 20 × 20 × 20 Å^3^, centered at X = 3.27Å, Y = 24.19Å, Z = 27.15Å, with a volume equal to 431.26 Å, and a discretization of 0.25 Å. The identification of the best-ranked lysozyme binding site was performed according to the details in the Methods section. Furthermore, the lysozyme flexibility properties from the 3D-molecular structure were evaluated from the whole lysozyme 3D-structure together with the corresponding Ramachandran crystallographic validation as depicted in Figure 5.

**Figure 6:**
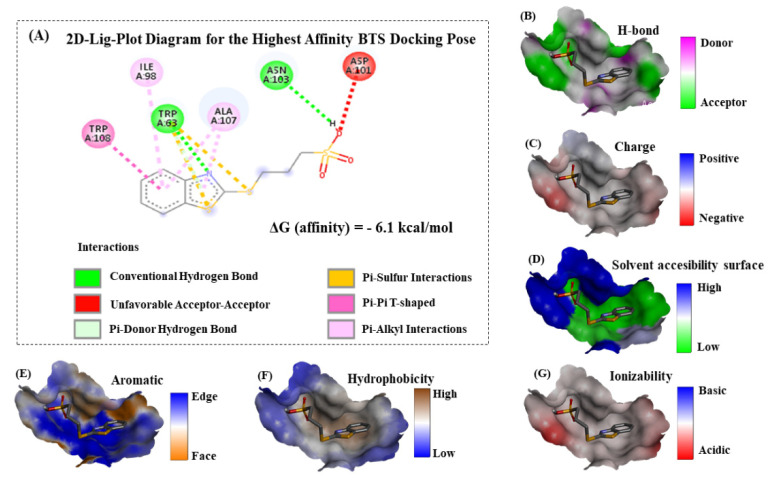
Inside the box, (**A**) represents the 2D-lig-plot diagram of interactions obtained for the best-ranked BTS docking pose with ΔG (docking affinity) = −6.1 kcal/mol for the BTS-lysozyme complex. Herein, dotted-lines represent different types of interactions with the corresponding color-labels represented below. Outside the box, (**B**–**G**) represent a breakdown of the different binding contributions to the BTS-lysozyme complex stability in the biophysical environment of interaction (i.e., lysozyme binding site) as: H-bond, electrostatic charge, solvent accessibility surface, aromatic interactions, hydrophobicity, and ionizability, all represented as Van der Waals surfaces with the corresponding bar-color of intensity, on the far right.

As can be seen in Figure 5, the accuracy of our theoretical docking approach was ensured by performing a proper prediction of the lysozyme binding-site, which suggests the best druggability for the BTS-ligand binding. In the present study, the prediction of the lysozyme-active binding site was performed using an *ezPocket* tool with a maximum score equal to 1.0 for the lysozyme. This binding pocket prediction method works with a consensus algorithm [40,41,42] which uses Delaunay triangulation to detect plausible lysozyme binding pockets, typically taking a few seconds and providing accurate Cartesian *XYZ*-coordinates for the docking box simulations. In this context, this computational task acquires a key relevance for exploring the molecular mechanisms of BTS interaction. For this study, the best-ranked binding-conformation that BTS adopts into the lysozyme-binding site was considered the one with the highest affinity according to the more negative values for the Gibbs free energy of binding (FEB) or ΔG (docking affinity). To explain these theoretical results, the 2D-Lig-Plot diagram for the docking complex formed by BTS-lysozyme can provide structural and mechanistic information on the simultaneous modulation of several lysozyme target-residues (i.e., effector and allosteric binding residues). This is accomplished by determining the contribution of the different types of non-covalent docking interactions and thermodynamics factors involved in the binding events, such as hydrophobic, H-bond, electrostatics, and aromatic Π-Π stacking interactions with its corresponding interatomic distances (*d_ij_*). For the best-ranked BTS binding-pose obtained from the BTS-lysozyme docking complex [43], see Figure 6.

According to the obtained results, we theoretically suggest that the BTS-ligand was able to interact in the predicted binding site placed in the middle of the lysozyme receptor following a spontaneous thermodynamics process according to the negative FEB values of binding ΔG (docking affinity) = −6.1 kcal mol^−1^ (or expressed in joules as 25.50 kJ mol^−1^). If we return to the ITC experimental results, we found a stoichiometry of one and a free energy of 26.58 kJ mol^−1^. It can be seen that both results support the theoretical predictions (in fact, the free energy quantities are really close), which reinforces the validity and quality of the results.

Particularly, we centered our attention on the fact that all the interactions detected correspond to non-covalent binding events. Herein, in order to tackle the different key contributions explaining the molecular mechanisms of interaction with lysozyme (see Figure 6A), we firstly identified a prevalence of aromatic hydrophobic Van der Waals interactions followed by conventional hydrogen bond interactions between the evaluated BTS-ligand and the lysozyme; namely, we had postulated on the basis of the thermodynamic values obtained by ITC. The aromatic pi-pi stacking edge-to-face interactions (a T-shaped fashion) are between the benzene ring belonging to the 2-(methylthio)-benzothiazole moiety of the BTS and the aromatic amino acid residue TRP108 of the lysozyme receptor. This type of interaction plays an important role in the stabilization of the BTS-lysozyme docking complex and was also verified by UV-vis and fluorescence measurements. Specifically, we identified a slightly displaced face-to-face (displaced pi-pi T-shaped) between the interacting aromatic benzene rings in the docking complex, and this theoretical observation can be corroborated in the Van der Waals map of interaction depicted in Figure 6E (please, note the presence of orange color locally distributed towards the region of the 2-(methylthio)-benzothiazole moiety in the Van der Waals map of interaction). In addition, these results fit very well with the hydrophobicity Van der Waals interaction map (Figure 6F) with a trend to express the prevalence of hydrophobic pi-pi T-shaped contacts with the presence of white-to-orange color locally distributed in the hydrophobicity map. Then, it is expected that the presence of the 2-(methylthio)-benzothiazole moiety of BTS in the lysozyme binding site can significantly affect properties such as solvent accessibility surface (Figure 6D) of the target aromatic amino acid residue TRP108 and its neighboring non-aromatic amino acid residues ALA107 and ILE98. Both interact with the 2-(methylthio)-benzothiazole moiety of BTS through pi-alkyl interactions in the lysozyme binding site (represented as light pink dotted lines in the lig-plot diagram in Figure 6), which strongly contributes to the stability of the formed docking complex (BTS-lysozyme). Furthermore, note that the pi-alkyl interactions involving the non-aromatic amino acid residue ALA107 is stronger than the ILE98, due to the presence of concomitant pi-donor hydrogen bond interactions with both planar aromatic moieties (benzene ring and heterocyclic 1,3-thiazole) from the 2-(methylthio)-benzothiazole moiety of BTS. In this instance, the pi-alkyl interaction is quite strong over the target residue ALA107 because it is well-known that the presence of concomitant pi-donor hydrogen bond contributes to a larger pi-electron delocalization. Furthermore, we identify the presence of another relevant interaction, as pi-sulfur interaction mixed with a conventional hydrogen bond shared between the lysozyme aromatic target residue (TRP63) with the sulfur group of the 1,3-thiazole moiety from the 2-(methylthio)-benzothiazole moiety of BTS-ligand. In this regard, it is important to note that conventional hydrogen bonds generally contribute to the stabilization of the docking complex (i.e., BTS-lysozyme) and aromatic pi-sulfur interaction buried at a lysozyme’s like TRP63 can affect the interaction energies of ring conformations in the lysozyme and can also modulate specific electron-donating/accepting substitutions on the aromatic ring from both lysozyme and the BTS ligand. Indeed, the presence of two additional interactions in the substitutions of the 2-(methylthio)-benzothiazole moiety of the BTS-ligand were identified in the BTS-lysozyme docking complex which involves electrostatic bonds with neighboring lysozyme target residues such as: (i) a thermodynamically favorable conventional hydrogen bond between the target residue ASN103, and (ii) a thermodynamically unfavorable acceptor-acceptor interaction between the target residue ASP101. Both modulate the electrostatic properties of the oxygen group belonging to the propanesulfonic moiety of the BTS-ligand which at the same time, modulates the binding properties of the 2-(methylthio)-benzothiazole moiety of BTS-ligand in the lysozyme binding site. The coexistence of different interactions could also be guaranteed from an experimental point of view, based on the loss of linearity that we observed in the Stern–Volmer plots (Figure 3A). In addition, the end result of this balance of interactions is that the complex formed is not excessively stable. The value of the binding constant obtained by ITC defined BTS as a moderate ligand.

The influence of these two last interactions from the electronic point of view can be well corroborated by the presence of a moderate behavior for the analyzed binding properties: acceptor/donor H-bond, charged interactions, and ionizability (as depicted in the Figure 6B,C,G, respectively), where a predominance of white-to-gray color locally distributed in both is observed, the propanesulfonic moiety of the BTS-ligand and the lysozyme receptor in the corresponding Van der Waals surface maps. Overall, the relevance of these types of interactions and specific orientations between aromatics, electrostatics, and the effect of substitution in the context of a lysozyme’s fold and function under the unbound and bound state is still unexplored and could have paramount significance in rational drug-design targeting lysozyme.

**Figure 7:**
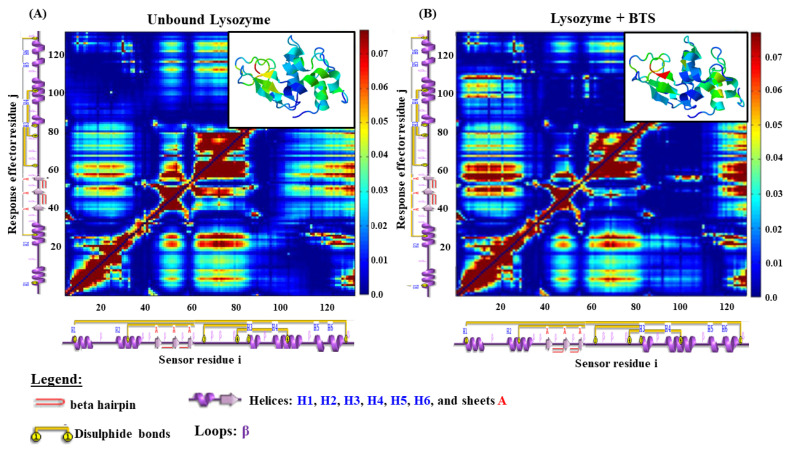
2D-Matrix representation-based local perturbation response scanning maps (LPRS maps) for the lysozyme receptor across the different conditions simulated. (**A**) LPRS map obtained for the unbound lysozyme receptor used here as a control simulation experiment, and (**B**) LPRS-maps obtained from the evaluated docking complex lysozyme plus BTS (representative of the bound state). On the right, the intensity bar color represents the i,j-lysozyme residue perturbations. Both LPRS-maps were performed by considering all the low-frequency normal modes to capture the global fluctuations associated with the lysozyme inter-residue network under unbound and bound states. In addition, in the top-right of each LPRS map, the 3D-structure of the lysozyme for both unbound and bound states is depicted just for visualization purposes of the structural differences according to local perturbations. Here transitions-based colored regions in the lysozyme whole structure (i.e., from dark blue to dark red) represent inter-residue distance perturbations in blocks of consecutive j-effector residues denoting conformational rigidity for large blocks of consecutively labelled-dark blue and conformational flexibilization for j-effector residues labelled-dark red under unbound and bound states. Regions labelled-light blue, green, and yellow represent moderate perturbations-based flexibility in the 3D-structure perturbation models.

Afterward, we examine how the BTS-ligand could affect the communication efficiency in the inter-residue network of the lysozyme receptor in the unbound and bound state (i.e., in the absence and presence of the BTS-ligand). To this end, we carried out an elastic network approach (ENM models) by regarding the local perturbations induced by the BTS-ligand in the C(α)-residue network that forms the lysozyme binding site [44,45,46]. In this instance, the local perturbations are propagated like an allosteric signal pathway across the communication network composed by C(α)-atom nodes which represent the residues and the network edges representing their mutual contacts connected by the two associated C(α)-residue nodes within a distance of 5.0 Å between C-alpha atoms in the lysozyme receptor structure. Herein, in the ENM model, the lysozyme binding residues composing the allosteric communication network are indexed by a non-ambiguous ordering. Then, using the ENM procedure allows for the visual representation of the strength of allosteric perturbation by generating the matrix of local perturbation response scanning maps from the aforementioned theoretical conditions (unbound and bound state of lysozyme) as depicted in Figure 7.

The obtained LPRS map approaches bring a structural and theoretical vision to the local perturbations induced by the BTS-ligand over the lysozyme receptor in the bound state, which remains unexplored until the present. In this sense, the local perturbations are defined by the collective anisotropic fluctuations generated from the intra-segment C*α*-C*α* atomic residue distances (labelled by the color intensity in the 2D-perturbation matrix) by the presence of a given ligand (i.e., the best-crystallographic binding pose of the BTS-ligand) which could affect the function and conformational properties of the binding residues of lysozyme. In this context, both the unbound and bound state are fully defined by the local perturbation response 3*N* × 3*N* Hessian matrix (*H_i,j_*) which describes the inter-residue allosteric communication pathway in the lysozyme [44,45,46]. Its *N*-elements (1 ≤ *i, j* ≤ *N*) are the second derivatives of the anisotropic network potential (V*_lysozyme_* or V*_lysozyme+ligand_*) which describes the (3*N*)-*XYZ*-displacements ($\Delta R_{ij}=R_{ij}-R_{ij}^{0}$) of all the (*i*,*j*)-residue pairs sensors (*i*) and effectors (*j*) in *N*-blocks of three dimensions each, and where *γ_R_* = *γ_L_* = 0.5 is an interaction constant, in the absence (lysozyme unbound state), and upon interactions (or perturbations as lysozyme plus BTS in the bound state) following the Equations (8) and (9):

(8) $$V_{\left(\mathrm{lysozyme}\right)}=\;\sum_{\left(i<j\right)\in N\left(R\right)}\frac{1}{2}\gamma_{R}{\left(R_{ij}-R_{ij}^{0}\right)}^{2}$$

(9) $$V_{\left(\mathrm{lysozyme}+\mathrm{BTS}\right)}=\;\sum_{\left(i<j\right)\in N\left(R\right)\cap N\left(L\right)}\frac{1}{2}\gamma_{R}{\left(R_{ij}-R_{ij}^{0}\right)}^{2}+\;\sum_{\left(i<j\right)\in N\left(R,L\right)}\frac{1}{2}\gamma_{L}{\left(R_{ij}-R_{ij}^{0}\right)}^{2}$$

Then, the anisotropic allosteric perturbations induced by the BTS-ligand in the inter-residue network of the lysozyme were determined using the LPRS maps, mainly focusing on the region of the previously predicted binding site containing around 120 residues. For the case of the control simulation represented by the lysozyme (unbound state), we show the patterns (i.e., theoretical physiological response) generated by coupled pairs of i-sensor residues vs. j-effector residues representing the native structural conformation of the lysozyme like a finely tuned allosteric signal transduction network; this models the catalytic function of the lysozyme binding-site. Herein, labelled-blue regions in the LPRS map denote conformational rigidity for large blocks of consecutive lysozyme i,j-target residues (perturbation direction i-sensor → j-effector residue pair) [44,45,46]. While on the opposite hand, labelled-yellow to dark-red regions in the LPRS map denote conformational flexibilization for large blocks of consecutive lysozyme i,j-target residues (perturbation direction i-sensor → j-effector residue pair).

According to the obtained results, we can clearly identify several differences in the LPRS maps between the unbound (Figure 7A) and bound state (Figure 7B) of the lysozyme. Particularly, we note that the first thirty i-sensor residues belonging to the α-helices (H1 and H2 connected by the first β-loop) are structurally and conformationally coupled with j-effector residues which exhibit moderate flexibilization to high conformational rigidity for large blocks of consecutive lysozyme (i-sensor → j-effector residues labelled-yellow to dark-blue color transition regions in the LPRS map of Figure 7A). This response is released in theoretical physiological conditions (lysozyme unbound state) that can be theoretically explained as physiological modulation or signal allosteric propagation toward j-effector residues belonging to the lysozyme sheets A (i.e., A1, A2, and A3), and also physiological modulation of the lysozyme j-effector residues forming the α-helices (H3, H4, H5, H6, and their corresponding β-loops). When we compare the same block of consecutive lysozyme i-sensor residues (first thirty) under a bound state (i.e., Figure 7B as LPRS map under interaction with the BTS-ligand forming the lysozyme-BTS docking complex) we found clear differences in the obtained perturbation response triggered by the lysozyme j-effector residues forming the α-helices (H3, H4, and their corresponding β-loops) which show that the BTS-interaction theoretically induces allosteric signal propagation, which strongly suggests an abnormal conformational flexibilization for large blocks of consecutive lysozyme j-effector residues (perturbation direction i-sensor → j-effector residue pair) at least for the lysozyme j-effector residues belonging to the α-helices (H3, H4, and their corresponding β-loops). Apparently, the BTS interaction does not induce long-distance allosteric perturbation in the lysozyme j-effector residues that form the α-helices H5, H6 and their corresponding β-loops which maintain their conformational properties (i.e., conformational rigidity) like the lysozyme unbound state. Concerning the block of consecutive i-sensor residues placed in the amino-acid sequence position from 60 to 115 (α-helices H3, H4, and their corresponding β-loops), we also detected a presence of conformational allosteric perturbations in the first twenty j-effector residues cluster induced under interaction with the BTS-ligand. These local perturbations were mainly based on moderate conformational flexibilization (labelled-dark yellow region) propagated from the cited i-sensor residues to the first twenty j-effector residues in the α-helices H1, H2, and its corresponding β-loop, in contrast with the LPRS map (unbound state) for the same region of j-effector residues, which presents an intrinsic conformational rigidity (labelled-yellow to dark-blue regions).

This theoretical observation has significant relevance because the block of consecutive lysozyme i-sensor residues placed amino acid sequence position from 60 to 115 of the lysozyme molecular structure includes the key interacting residues previously identified in the 2D-lig-plot analysis (i.e., TRP63, ILE98, ASP101, ASN103, ALA107, and TRP108) which allows for the corroboration and validation of the influence of the non-covalent binding events between the BTS-ligand with the lysozyme receptor.

## 3. Materials and Methods

### 3.1. Reagents

Lysozyme from chicken egg white, (lyophilized powder, protein ≥ 90%, ≥40,000 units/mg), 3-(2-Benzohiazolylthio)-1-propane-sulfonic acid sodium salt, and C_10_H_10_NNaO_3_S_3_ (BTS, MW = 311.38 g·mol^−1^, 95%) were purchased from Sigma-Aldrich and used without further purification. Samples were freshly prepared for each experiment within 1 h. prior to usage. Tripe-distilled and degassed water was used for the preparation of the respective aqueous solutions.

### 3.2. Isothermal Titration Calorimetry

Experiments were performed at a temperature of 298.15 K using a VP-ITC microcalorimeter (MicroCal Inc., Northampton, MA, USA) [47]. To determine the binding parameters, BTS solutions (0.5 mM) were introduced into a syringe (296 μL), while the sample cell (1.4166 mL) was filled with the macromolecule solutions (Lysozyme 0.02 mM). In order to assure homogeneity, stirring was maintained at 416 rpm throughout the entire test. Equilibrium time before each experiment was about one hour, sufficient to assure the power base line stabilization. Twenty-six injections of 10 μL at a constant rate of 0.5 μL s^−1^ were added every 500 s. To guarantee that the signal was not affected by overcompensation mechanisms and to eliminate negative signals, a reference power of 15 μJ s^−1^ was applied. Dilution experiments of pure lysozyme were also carried out. Obtained data were subtracted from those measured for the ligand-protein systems, assuring that all the heat produced in the cell was due solely to the binding process.

### 3.3. Circular Dichroism

Far-UV circular dichroism (CD) spectra were obtained using a JASCO-715 automatic recording spectropolarimeter (Japan) with a JASCO PTC-343 Peltier-type thermostated cell holder. Quartz cuvettes with a 0.2-cm pathlength were used. CD spectra of pure proteins and proteins-BTS dilute solutions were recorded from 190 to 270 nm. Protein concentration was 0.5 mg/mL and BTS concentrations varied from 0.1 to 0.5 mM. The following settings were used: resolution, 1 nm; bandwidth, 1 nm; sensitivity, 50 mdeg; response time, 8 s; accumulation, 3; and scan rate 50 nm/min. Corresponding absorbance contributions of buffer solution and doubly distilled water were subtracted with the same instrumental parameters. Data were reported as molar ellipticity and determined as [θ]_λ_ = θ_λ_M_r_/ncl, where c is the protein concentration, l is the path length of the cell, θ_λ_ is the ellipticity given by the instrument at a wavelength λ, M_r_ is the molecular mass of the protein, and n is the number of residues. The measured CD curves are a superposition of the individual spectra of α -helix, β-sheet, β-turn, and randomly coiled conformations. The secondary structure content was analyzed by Dichroweb program using the CONTIN algorithm.

### 3.4. UV-Vis Absorption Spectra

UV-vis absorption spectra were recorded using a Cary 100 Bio UV-Vis Spectrophotometer. The spectral range studied was 240–400 nm. Lysozyme concentration was maintained constant at 0.02 mM and increasing concentrations of BTS from 0 mM to 0.01 mM were added to study how the different ratios affected the spectral results.

### 3.5. Fluorescence Measurements

Fluorescence measurements were conducted on a Cary Eclipse spectrofluorometer. The excitation and emission splits were 5 nm. The synchronous fluorescence spectra were recorded by adjusting the data interval at 1 nm and the average time at 0.5 s. The measured range was 250–450 nm. Upon excitation at 280 nm, the fluorescence spectra of lysozyme-BTS systems were recorded at 298.15 K. Protein concentration was fixed at 0.02 mM and increasing concentrations of drug were added, from 0 mM to 0.20 mM. With the purpose of avoiding inaccurate results, inner filter effects were corrected for the quenching experiments by using the following expression: F_corr_ = F_obs_ · e ^ [(A_exc_ + A_em_)/2]; where A_exc_ and A_em_ are the absorptions of the systems at the excitation and the emission wavelength, and F_corr_ and F_obs_ are the corrected and observed fluorescence intensities, respectively. For data processing, UV-Vis-IR Spectral Software (FluorTools) was used [48].

### 3.6. Performing Molecular Docking Simulation

To tackle the binding interactions between BTS and lysozyme, an in silico mechanistic study based on molecular docking was applied. To this end, first, we prepared the lysozyme receptor file, which was stored in the *RCSB Protein Data Bank* (PDB) X-ray structures, i.e., with *PDB ID*: 1HER [49,50]. Afterward, the lysozyme molecular structure was optimized by applying the AutoDock Tools 4 software [51,52]. This procedure starts removing all the crystallographic waters as well as all co-crystallized ligands, if any. Next, H-atoms were included in the lysozyme 3D-structure, following an appropriate hybridization geometry adding partial atomic charges and setting the protonation states of the X-ray lysozyme.pdb model [51]. Following this, the BTS ligand was retrieved from the Pubchem Data Base Chemical Structure Search (PubChem CID: 162569; MF: C_10_H_11_NO_3_S_3_) [53]. The BTS geometry optimization was performed by applying the MOPAC extension based on NDDO approximation [54]. To explore the BTS docking mechanisms with lysozyme, we used the Vina scoring function developed by Trott et al. [52] to achieve the free energy of binging (FEB, kcal/mol) which approximates the standard chemical potentials. Next, before the docking approach, the lysozyme binding-sites were predicted through the ezPocket tool for binding-site identification [40,41]. This step was carried out by delimiting the access to lysozyme-cavities, as Van der Waals surfaces are likely to bind to the BTS. Herein, the ezPocket tool applies Delaunay triangulation with weighted points to predict plausible binding sites in a given receptor [42]. Then, the volumetric map of the lysozyme binding-site was generated jointly to the Cartesian *XYZ*-coordinates for the docking box simulations [55], i.e., a grid box size with dimensions of X = 20Å, Y = 20Å, Z = 20Å, and a grid box center X = 3.27Å, Y = 24.19Å, Z = 27.15Å, with a volume equal to 431.26 Å^3^. Afterward, the docking accuracy was fixed at 50 and after that, the best nine BTS conformational binding poses were selected [52]. Lastly, the docking affinity (i.e., FEB values for the obtained docking complexes) were categorized as energetically-unfavorable when the FEB of lysozyme-BTS complexes ≥ 0 kcal/mol, thus indicating either extremely low or complete absence of docking affinity. Otherwise, the lysozyme-BTS docking complexes were categorized as medium to high docking affinity.

### 3.7. 2D Lig-Plot Diagrams

This method was carried out to evaluate the influence of the FEB values for the different contributions of the BTS binding-poses interacting with the lysozyme receptor. Here, to obtain the relevant intermolecular interactions between BTS poses with the lysozyme receptor, 2D Lig-Plot diagrams were obtained just considering the best-ranked BTS docking pose. To tackle this objective, a software named Discovery Studio was used. This software is able to determine the non-covalent intermolecular interactions present in a given lysozyme-BTS complex and it automatically builds a 2D-interaction diagram that includes hydrophobic, H-bond, electrostatics, and Π-Π stacking interactions and its corresponding interatomic distances (*d_ij_*) for each BTS binding-poses from the obtained docking complexes [43].

### 3.8. Performing Perturbation Response Maps

This computational approach allows for the evaluation of the degree of conformational change (i.e., interatomic distance perturbation between residue fluctuations) induced by a given ligand (i.e., BTS) in the residues network (lysozyme binding site) by describing the receptor-ligand complex (Lyso*_(j)_-BTS_(i)_*or* R-L*) interaction potential (*U*) as a Hookean potential using an elastic network model (ENM) [44,45,46,56,57]. To this end, the perturbation interaction-induced by the best ranked BTS docking pose on the lysozyme binding-site was estimated by averaging all the lysozyme binding-residues from different amplitudes between the perturbed (bound state) and the unperturbed state (unbound state) of the lysozyme structure regarding the displacements from equilibrium using anisotropic vibrational analysis [46] following the equation:

(10) $$e_{p_{\left(i\right)}}=\frac{1}{N_{a}}\sum_{j=1}^{N_{a}}\left|p_{j}-u_{j}\right|$$

where $e_{p_{\left(i\right)}}$ represents the effect of the perturbation response in the anisotropic normal mode (*i*), *p_j_* is the displacement of the fibrinogen lysozyme residue (*j*) in the perturbed normal mode (${\left(d_{ij}-d_{ij}^{0}\right)}^{2}$_R-L_), *u_j_* represents the displacement of individual lysozyme interacting residues (*j*) in the unbound or unperturbed normal mode (${\left(d_{ij}-d_{ij}^{0}\right)}^{2}$_R-R_), and *N_a_* is the number of binding site residues obtained from the 2D Lig-Plot diagram analysis for the best-ranked BTS binding-pose in the lysozyme receptor. The $e_{p_{\left(i\right)}}$ perturbation effect is depicted as a 2D-matrix represented by a local perturbation response scanning map (LPRS map) for the best-ranked BTS binding-pose. Here the *i*th rows are related to the response generated upon perturbing the lysozyme residue (*i*) and its average (i.e., overall *k*-receiver’s residues of lysozyme), while the *k*th columns of the LPRS maps depict the sensitivity in response to the perturbation for all the lysozyme allosteric residues (*j*-sensors residues).

## 4. Conclusions

In this work, by combining experimental and computational methods, the formation of the lysozyme BTS complex has been accurately and rigorously characterized. The combined use of the two approaches has allowed us not only to corroborate the results but also to complement them. In this way, it has been shown that the complex stoichiometry is one-to-one due to the balance of hydrophobic and hydrogen bond interactions and the BTS acts as a moderate binder. Circular dichroism results showed that the changes in the secondary structure of the protein, especially at low concentrations, are relatively slight, which could mean that its function is not altered. However, perturbation methods revealed that BTS induces allosteric signal propagation which strongly suggests non-physiological conformational flexibilization in large blocks of lysozyme residues affecting the α-helices. This fact highlights the great impact that slight conformational changes can have on the functionality of proteins. In this way, we have shown that combining theoretical and experimental approaches provides the broad and precise approach that allows us to delve deeper into this type of system.

## Figures and Tables

**Table 1:** Contents (in %) of the α-helices and β-sheets elements in lysozyme as a function of BTS concentration [21].

[BTS] (mM)	α-Helix	β-Sheet
0.00	19	56
0.05	16	36
0.10	17	48
0.50	12	47

## Sample Availability

The samples of compounds are not available from authors.
